# Effect of electroacupuncture treatment on functional constipation in adults: A protocol for systematic review

**DOI:** 10.1097/MD.0000000000024870

**Published:** 2021-03-12

**Authors:** Na Li, Ruihui Wang, Xia Ai, Xinrong Guo, Juan Liu, Dong Wang, Lei Sun, Rongchao Zhang

**Affiliations:** a School of Acupuncture-Tuina, Chengdu University of Traditional Chinese Medicine, Chengdu, Sichuan; b School of Acupuncture-Tuina, Shaanxi University of Traditional Chinese Medicine, Xi’an, Shaanxi, China.

**Keywords:** electroacupuncture, functional constipation, protocol, randomized controlled trials, systematic review

## Abstract

**Background:**

Electroacupuncture has been widely used to treat functional constipation. But its efficiency has not been scientifically and methodically evaluated. The objective of this study is to evaluate the efficiency and safety of the electroacupuncture treatment for functional constipation in adults.

**Methods:**

This protocol of systematic review will be conducted in accordance with the Preferred Reporting Items for Systematic Review and Meta-analysis Protocols (PRISMA-P). We will conduct the literature searching in the following electronic databases: the Cochrane Library, MEDLINE, EMBASE, Web of Science, Springer, the Chinese Science Citation Database (CSCD), China National Knowledge Infrastructure (CNKI), the Chinese Biomedical Literature Database (CBM), Wanfang, and the Chinese Scientific Journal Database (VIP). The time limit for retrieving studies is from establishment to July 2020 for each database. All published randomized controlled trials (RTCs) related to this review will be included. Review Manager (V.5.3.5) will be implemented for the assessment of bias risk and data analyses. The selection of the studies, data abstraction, and validations will be performed independently by 2 researchers.

**Results:**

This review will assess the clinical efficacy and safety, as well as the acupoints characteristics of electroacupuncture on functional constipation (FC) in adults.

**Conclusion:**

This review will summarize the current evidence of electroacupuncture on FC outcomes and provide guidance for clinicians and patients to select electroacupuncture for FC in adults.

**Trail registration number:**

This protocol of systematic review has been registered on PROSPERO website (No. CRD42019146715).

## 1 Introduction

### 1.1 Description of the condition

Functional constipation (FC) is a common functional gastrointestinal disorder (FGID) without any physiological or anatomical abnormalities, and is diagnosed according to the Rome criteria, which manifests with infrequent bowel movements, hard/lumpy stools, excessive straining, and/or incomplete evacuation feelings.^[1–3]^ The global prevalence of FC is 1.9% to 27%, with 17.1% in Europe, 15.3% in Oceania, and 1.9% to 27.2% in North America.^[4,5]^ A new epidemiological survey of the United States, Canada, and the United Kingdom shows that the incidence of FC in adults is 7.8%.^[6]^

The incidences of FC also negatively impact the quality of life of the individuals, loss of productivity due to emotional distress, and economic burden for a person seeking treatment. The direct cost of constipation ranges from $1912 to $7522 per year per patient.^[7–9]^ Traditionally, treatment of FC in adults involves lifestyle interventions, pelvic floor interventions, and pharmacological therapy such as osmotic laxatives; bulking agents, and stimulant laxatives; however, approximately half of patients are dissatisfied with these treatment strategies because of their limited efficacy and there are few long-term clinical studies on their effects.^[10–12]^

Electroacupuncture therapy is an external therapy used in traditional Chinese medicine. By adding external electrical stimulation to traditional acupuncture, the stimulus gets doubled and the therapeutic efficacy gets enhanced accordingly. It is an emerging therapy that combines acupuncture with electrical stimulation.^[13]^ Nowadays, electroacupuncture has been widely used clinically by doctors of traditional Chinese Medicine, gastroenterology department to treat FC with satisfied efficacy.^[14–18]^ However, no relevant review or protocol has been published to date. Therefore, it is necessary to conduct evidence-based review to evaluate the efficacy and safety of electroacupuncture for FC in adults. It is urgently needed to accomplish this review.

## 2 Methods

The protocol for this review has been registered in the International Prospective Register of Systematic Reviews (PROSPERO) (registration number: CRD42019146715) on 16 December, 2019. Available online: https://www.crd.york.ac.uk/prospero/#myprospero. The protocol will be strictly developed under the guidelines of the Preferred Reporting Items for Systematic Reviews and Meta-Analyses protocols (PRISMA-P)^[19]^ and Cochrane Handbook for Systematic Reviews of Interventions.^[20]^

### 2.1 Selection criteria

#### 2.1.1 Types of studies

As the randomized controlled trials (RCTs) are reliable and feasible, RCTs will only be included. After the research, published clinical trials which reported the efficacy and safety on electroacupuncture for FC will be included. Literature of animal research, case reports, reviews, meta-analyses, retrospective studies, and non-RCTs will be excluded.

#### 2.1.2 Types of patients

Patients aged ≥18 years and diagnosed with FC according to ROME II, III, or IV criteria will be included, without limits on gender, age, race, nationality, and medical units.

#### 2.1.3 Types of interventions and comparisons

We will include the studies using electroacupuncture as the sole intervention in the experimental group, while we have no restrictions on intervention in the control group. Studies involving electroacupuncture combined with other therapies will be included if the other therapies are used equally in both THE experimental and control groups.

#### 2.1.4 Types of outcomes

The main outcomes of this review include the mean weekly complete spontaneous bowel movements (CSBMs). Additional outcomes of this review include mean weekly spontaneous bowel movements (SBMs), mean scores for stool consistency and straining of SBMs, and Patient Assessment of Constipation Quality of Life questionnaire (PAC-QOL score).

### 2.2 Search methods for the identification of studies

#### 2.2.1 Electronic search strategy

The electronic databases of the Cochrane Library, MEDLINE, EMBASE, Web of Science, Springer, the Chinese Science Citation Database (CSCD), China National Knowledge Infrastructure (CNKI), the Chinese Biomedical Literature Database (CBM), Wanfang, and the Chinese Scientific Journal Database (VIP) will be searched from the establishment to July 1st, 2020. All published RCTs on this subject will be included. Exemplary search strategy of MEDLINE is listed in Table 1, and terms are conformed to the medical subject heading. According to the different retrieval modes, keywords may combine with free words and comprehensive search will be performed.

**Table 1:** Search strategy used in MEDLINE database.

#1 Title/Abstract: electroacupuncture
#2 Title/Abstract: electrical acupuncture
#3 Title/Abstract: (electric AND (acupuncture OR needle OR acupoint OR point OR stimulate))
#4 #1 OR #2 OR #3
#5 Title/Abstract: constipation
#6 Title/Abstract: functional constipation
#7 Title/Abstract: chronic constipation
#8 Title/Abstract: chronic functional constipation
#9 Title/Abstract: chronic severe functional constipation
#10 Title/Abstract: functional gastrointestinal disorder
#11 Title/Abstract: functional defecatory disorder
#12 Title/Abstract: FC
#13 Title/Abstract: CC
#14 Title/Abstract: CSFC
#15 #5OR #6 OR#7 OR#8 OR#9 OR#10 OR#11 OR#12 OR#13#14
#16 Title/Abstract: randomized controlled trial
#17 Title/Abstract: controlled clinical trial
#18 Title/Abstract: randomized
#19 Title/Abstract: randomly
#20 Title/Abstract: RCT
#21 Title/Abstract: trial
#22 #16 OR#17 OR#18 OR#19 OR#20 OR#21
#23 Title/Abstract: ≥18 yr of age
#24 Human
#25 #4 AND #15 AND #22 AND #23AND#24

FC = functional constipation, RCT = randomized controlled trial.

Relevant keywords were used to create search strategies, as listed in Table 1. In the selection process, only research conducted in humans will be included in further review.

The MEDLINE search strategy in Table 1 will be adapted for other databases.

### 2.3 Data extraction, quality, and validation

#### 2.3.1 Study inclusion

**Figure 1:**
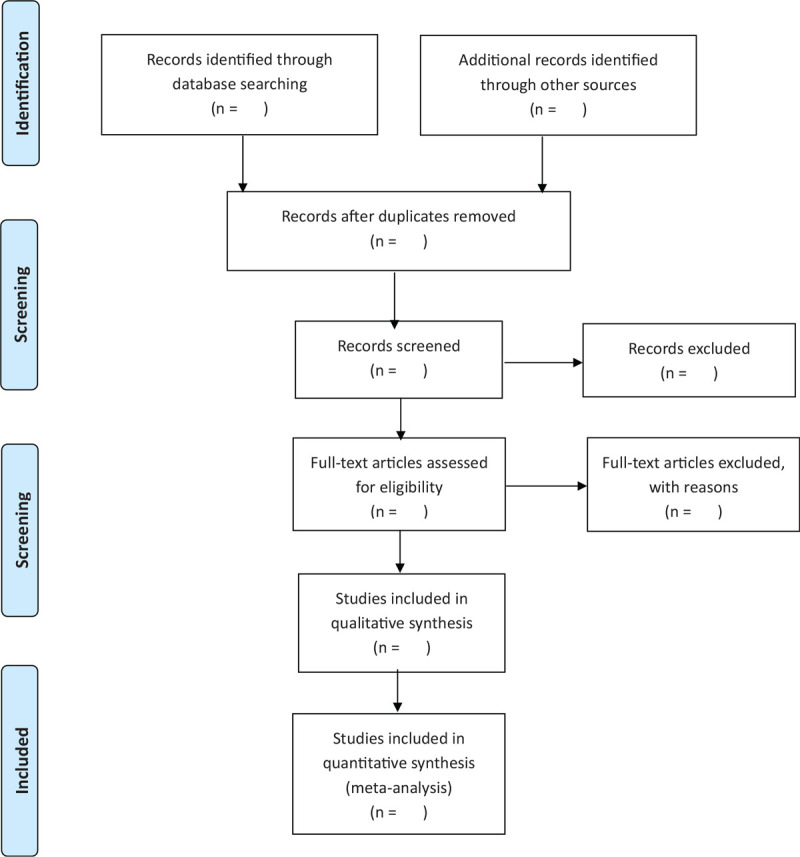
PRISMA flow diagram of study process. PRISMA = Preferred Reporting Items for Systematic Reviews and Meta-Analyses

Two reviewers (NL and XA), who will be told the aim and process of the system review, will select the trials and studies independently according to the criteria for inclusion by reading the titles and abstracts; if necessary, the full text will be read for further assessment. The discrepancies in the process will be discussed and solved using RHW. Details of the research choices are shown and exclusive studies will be listed and explained. The process of study selection will be presented in Preferred Reporting Items for Systematic Reviews and Meta-Analyses (PRISMA) flow diagram (Fig. 1). To ensure consistency, we will perform calibration exercises on methodological steps of the review process before assessment.

#### 2.3.2 Data extraction and management

An electronic form will be established via Excel to extract substantial contents, and then filled by 2 reviewers independently. Information was extracted from the qualified articles by using a standardized data extraction form as follows: authors’ name, year of publication, country, study design, age and gender of patients, intervention, sample size, outcomes, adverse events, and follow-up. Disagreements will be solved by group discussion or consulting seniors. However, if we fail to reach a consensus, the authors of trials will be contacted for further details and verification.

#### 2.3.3 Managing missing data

If any data information is not sufficient in the included trials, we will try to contact the first or corresponding author by email or phone, requesting adequate information and details of the studies included to retrieve missing or insufficient trial data. However, if the author is not available or sufficient information cannot be obtained, we will have a group discussion and analysis based on the current information. Meanwhile, the potential impact of missing data will be taken into account, and a relative discussion will be presented in the result section.

#### 2.3.4 Assessment of bias risk and quality of the included studies

Two independent authors assess the risk of bias of each included article according to the Cochrane Handbook for Systematic Reviews of Interventions. The methodological quality will be evaluated from the following domains: random sequence generation, allocation concealment, blinding of participants and therapists, blinding of outcome assessment, incomplete outcome data, selective reporting, and other biases. The judgements on these items will be categorized as “low risk of bias,” “high risk of bias,” or“ unclear risk of bias.” Discrepancies will be resolved by negotiation or by consulting other reviewers.

#### 2.3.5 Measures of treatment effects

Dichotomous data and continuous variables are included in the outcomes of interest, and we will use the risk ratio (RR) to express dichotomous data and use mean difference (MD) to assess differences in the continuous outcomes between the groups. Although different methods for the measurement of outcomes are used in different trials, we will choose standardized mean difference (SMD) if they have the same outcomes. The corresponding 95% confidence interval (CI) for each parameter will be calculated between the electroacupuncture treated group and the control group. We will choose a descriptive review if quantitative synthesis is not appropriate.

#### 2.3.6 Assessment of heterogeneity

The research will be performed with RevMan5.3.5 software. *P* < .5 will be defined as statistically significant between studies. The heterogeneity of studies will be evaluated by *I*^2^ statistic. The following criteria will be used: *I*^2^ < 50% will be deemed as low heterogeneity; *I*^2^ between 50% and 75% will be considered as moderate heterogeneity; *I*^2^ > 75% will be considered as high heterogeneity.

#### 2.3.7 Subgroup analysis

When heterogeneity is high, we will perform a subgroup analysis based on different controls, intervention time, treatment frequency, follow-up duration, and outcome measurements. We will also tabulate the adverse reactions and then perform an evaluation.

#### 2.3.8 Sensitivity analysis

If possible, a sensitivity analysis will be performed to verify the robustness of the review conclusions. The impact of methodological quality, sample size, and missing data will be assessed. In addition, the analysis will be repeated after the exclusion of low methodological quality studies.

## 3 Discussion

FC is one source of distress. This disease has brought physical, psychological, and economic burdens to many people in many countries and has brought great suffering. It has aroused more and more concern all over the world. Electroacupuncture (EA), derived from the integration of traditional acupuncture and modern electrical stimulation, is another type of acupuncture. Now, EA has become more and more widely used in FC because it is a relatively straightforward, safe, and cheap therapy compared with other conventional therapies. But at the present, there is no systematic evaluation report on its therapeutic effectiveness and safety for the treatment of FC.

The protocol of this systematic review study aims to assess the efficacy, safety, and cost benefits of acupuncture in the treatment of FC in adults. Meanwhile, we have tried our best to search and found that no relevant systematic review and meta-analysis concerning this topic has been reported in the last 5 years, and we will integrate the latest and most comprehensive clinical evidence in this field, hoping to offer a greater variety of treatment options for patients with FC and inspire more peer experts and doctors to carry out as many relevant studies as possible in the future.

## Author contributions

**Conceptualization:** Na Li, Ruihui Wang.

**Data curation:** Na Li, Xia Ai, Xinrong Guo, Juan Liu, Dong Wang, Lei Sun, Rongchao Zhang.

**Investigation:** Xinrong Guo.

**Methodology:** Na Li, Xia Ai.

**Validation:** Ruihui Wang.

**Visualization:** Na Li.

**Writing – original draft:** Na Li, Xia Ai.

**Writing – review & editing:** Na Li, Ruihui Wang, Lei Sun.
